# Neuroprotective effects of oleic acid in rodent models of cerebral ischaemia

**DOI:** 10.1038/s41598-019-47057-z

**Published:** 2019-07-24

**Authors:** Jungbin Song, Young-Sik Kim, Dong Hwan Lee, Sung Hyun Lee, Hyo Jin Park, Donghun Lee, Hocheol Kim

**Affiliations:** 1 Department of Herbal Pharmacology, College of Korean Medicine, Kyung Hee University, 26 Kyungheedae-ro, Dongdaemun-gu, Seoul 02447 Republic of Korea; 2 Korea Institute of Science and Technology for Eastern Medicine (KISTEM) NeuMed Inc., 88 Imun-ro, Dongdaemun-gu, Seoul 02440 Republic of Korea; 3 Department of Herbal Pharmacology, College of Korean Medicine, Gachon University, 1342 Seongnamdae-ro, Sujeong-gu, Seongnam-si, Gyeonggi-do 13120 Republic of Korea

**Keywords:** Brain injuries, Stroke

## Abstract

Oleic acid (OA) is released from brain phospholipids after cerebral ischaemia; however, its role in ischaemic injury remains unknown. We hypothesised that OA has neuroprotective effects after cerebral ischaemia, which may be exerted through peroxisome proliferator-activated receptor gamma (PPAR-γ) activation, since OA is an endogenous ligand of PPAR-γ. The effects of OA administration were evaluated in rodent models of middle cerebral artery occlusion (MCAO), photothrombosis, and four-vessel occlusion (4-VO). We determined the time window of therapeutic opportunity and examined the ability of the PPAR-γ antagonist GW9662 to reverse OA’s protective effects after MCAO. We found that OA administration decreased the MCAO-induced infarct volume and functional deficits, photothrombosis-induced infarct volume, and 4-VO-induced hippocampal neuronal death. Additionally, OA was highly efficacious when administered up to 3 h after MCAO. Pre-treatment with GW9662 abolished the inhibitory effects of OA on the infarct volume and immunoreactivity of key inflammatory mediators in the ischaemic cortex. Our results indicate that OA has neuroprotective effects against transient and permanent focal cerebral ischaemia, as well as global cerebral ischaemia. It may have therapeutic value for the ischaemic stroke treatment with a clinically feasible therapeutic window. The OA-mediated neuroprotection might be attributable to its anti-inflammatory actions through PPAR-γ activation.

## Introduction

Oleic acid (C18:1, *cis*-9) is the most abundant monounsaturated fatty acid in dietary fats and oils. In the brain, it is a major constituent of membrane phospholipids and is highly concentrated in myelin^1,2^. Recent studies have demonstrated that oleic acid is necessary for the appropriate development and functioning of the brain. During brain development, oleic acid is used to synthesise myelin phospholipids^3^ and acts as a neurotrophic factor by promoting axonal and dendritic growth, enhancing neuronal migration and aggregation, and facilitating synapse formation^4–7^. A significant decline in oleic acid has been found in the brains from patients with Alzheimer’s disease and major depressive disorder compared to normal brain tissue^8,9^. Oleic acid has been shown to reduce amyloidosis in *in vitro* and rodent models of Alzheimer’s disease^10^ and to alleviate the toxic effects of 7-ketocholesterol, a lipid peroxidation product that is increased in patients with neurodegenerative diseases^11,12^.

Under conditions of cerebral ischaemia, free fatty acids are rapidly released from brain membrane phospholipids by the activation of phospholipase and accumulate within a few minutes after onset^13^. Regarding the role of fatty acids in cerebral ischaemia, more attention has been given to omega-3 and -6 polyunsaturated fatty acids (PUFAs) and less to the other fatty acids. The omega-3 PUFAs, such as docosahexaenoic acid (C22:6), eicosapentaenoic acid (C20:5), and α-linolenic acid (C18:3), have been well established to reduce neuronal cell death following cerebral ischaemia^14,15^, whereas the role omega-6 arachidonic acid plays in neuroprotection is rather controversial according to the literature^16,17^. Another omega-6 PUFA, linoleic acid (C18:2), has been reported to be involved in ischaemic brain damage through its oxidised metabolites that regulate neurotransmission^18^. However, despite the substantial accumulation of oleic acid in the ischaemic brain^19^, its role in the pathogenesis of cerebral ischaemia remains unknown.

Available evidence suggests that oleic acid may protect against ischaemic brain damage. We previously reported the neuroprotective effects of the flower buds of *Buddleja officinalis* in a focal cerebral ischaemia rat model^20^ and identified oleic acid in the active fraction of *B*. *officinalis* extracts (data not published). An *in vitro* study demonstrated that oleic acid attenuates the microglial inflammatory responses that promote neuronal death after cerebral ischaemia^21,22^. Oleic acid is an endogenous agonist of peroxisome proliferator-activated receptor gamma (PPAR-γ), a ligand-activated transcription factor belonging to the nuclear receptor superfamily^23^. In addition to its well-established role in regulating glucose and lipid metabolism, PPAR-γ has been suggested as a therapeutic target for neuroprotection in cerebral ischaemia^24^. It has been demonstrated that the administration of PPAR-γ agonists provides neuroprotection and improves neurological functions in animal models of cerebral ischaemia^25–28^. Based on these findings, we hypothesised that oleic acid has neuroprotective effects in cerebral ischaemia and that these effects might be exerted via PPAR-γ activation.

In the present study, we tested these hypotheses by investigating the ability of systemic treatment with oleic acid to reduce neuronal injury after cerebral ischaemia. Cerebral ischaemia can be transient (followed by reperfusion) or permanent, and the lack of blood flow can affect a specific brain region or widespread areas. Since there is no single experimental model that mimics all of these clinical aspects, we examined the neuroprotective effects of oleic acid in three different rodent models relevant to each of these conditions, as follows: transient focal ischaemia (middle cerebral artery occlusion [MCAO] model), permanent focal ischaemia (photothrombosis model), and transient global cerebral ischaemia (four-vessel occlusion [4-VO] model). We further evaluated the behavioural outcomes and determined the therapeutic time window after transient focal cerebral ischaemia. To elucidate whether PPAR-γ activation was required for oleic acid-mediated protection in cerebral ischaemia, we examined the ability of the PPAR-γ antagonist GW9662 to reverse the neuroprotective effects of oleic acid.

## Results

### Effects of oleic acid on infarct volume and functional deficits after MCAO

**Figure 1:**
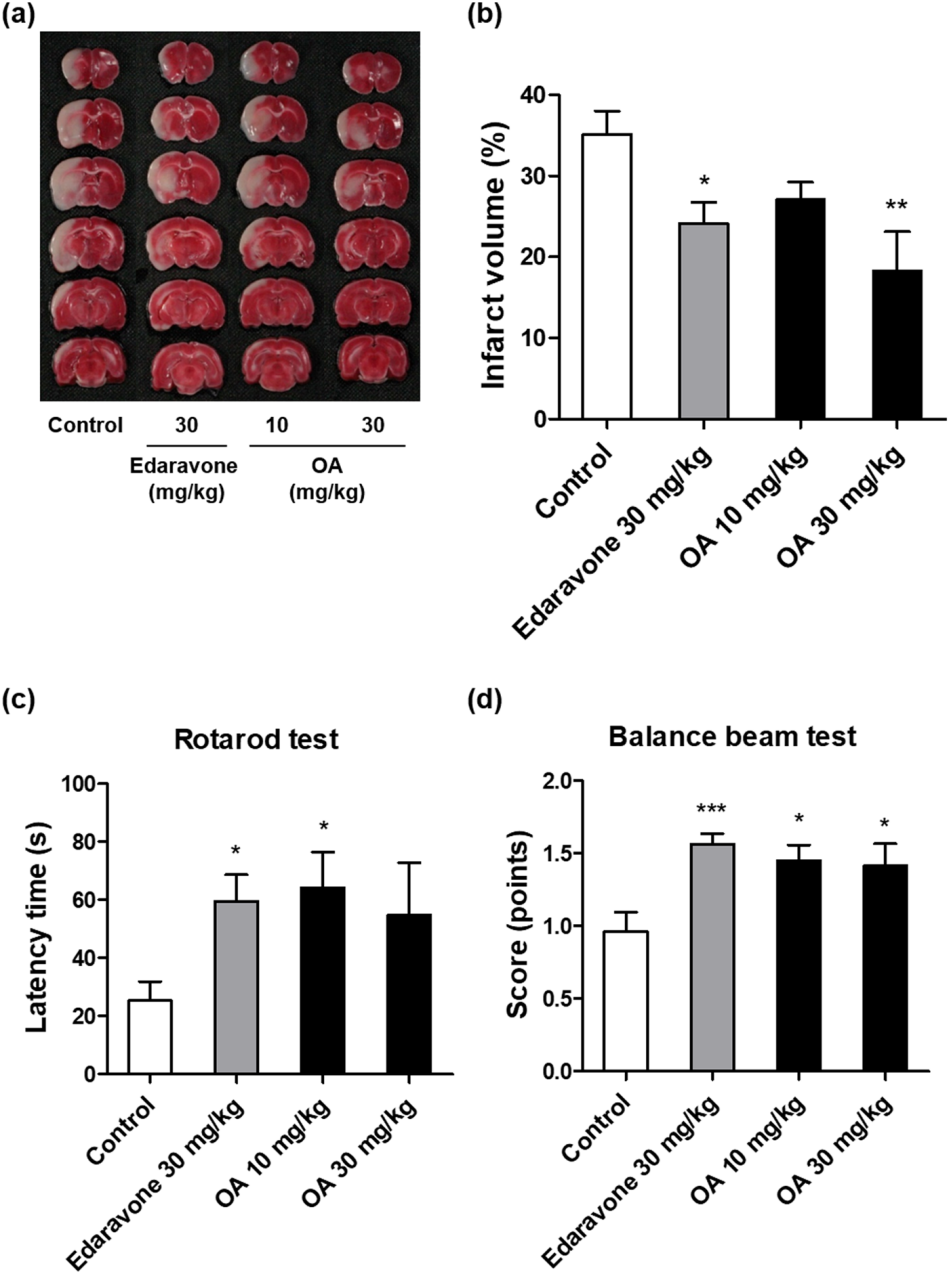
Effects of oleic acid (OA) on infarct volume and functional outcomes after middle cerebral artery occlusion in Sprague-Dawley rats. (**a**) Representative 2,3,5-triphenyl tetrazolium chloride-stained coronal sections of rat brain. The viable tissue is stained deep red, whereas the infarcted tissue is unstained. (**b**) Quantification of infarct volume. (**c**) Latencies to fall off the rotarod. (**d**) Balance beam scores. *p < 0.05, **p < 0.01, and ***p < 0.001 vs. the vehicle-treated control group. Values are the mean ± the standard error of the mean (n = 13, 17, 7, and 8 for the control, edaravone, OA 10 mg/kg, and OA 30 mg/kg groups, respectively).

Transient MCAO induced focal lesions in the frontal, parietal, and temporal cortices, and caudate putamen, which are limited to the MCA territory, after 24 h in Sprague-Dawley (SD) rats (Fig. 1a). Compared to the control group, which had an infarct volume of 35.1 ± 2.9%, rats treated with oleic acid at doses of 10 and 30 mg/kg (intraperitoneally) had reduced infarct volumes of 27.1 ± 2.1% (not significant) and 18.3 ± 4.8% (*p* < 0.01), respectively (Fig. 1b). The positive drug, edaravone (30 mg/kg, intraperitoneally), also significantly reduced the infarct volume to 24.1 ± 2.6% (*p* < 0.05). In the rotarod test, oleic acid prolonged the latency time from 25.4 ± 6.6 s (control group) to 64.3 ± 12.2 s (10 mg/kg-treated group, *p* < 0.05) and 54.9 ± 17.9 s (30 mg/kg-treated group) (Fig. 1c). The oleic acid 10 mg/kg-treated (1.5 ± 0.1 points, *p* < 0.05) and 30 mg/kg-treated (1.4 ± 0.2 points, *p* < 0.05) groups had significantly better balance beam scores than did the control group (1.0 ± 0.1 points) (Fig. 1d). The administration of edaravone (30 mg/kg, intraperitoneally) also significantly improved the rats’ performances on the rotarod and balance beam tests.

### Effects of oleic acid on infarct volume after photothrombosis

**Figure 2:**
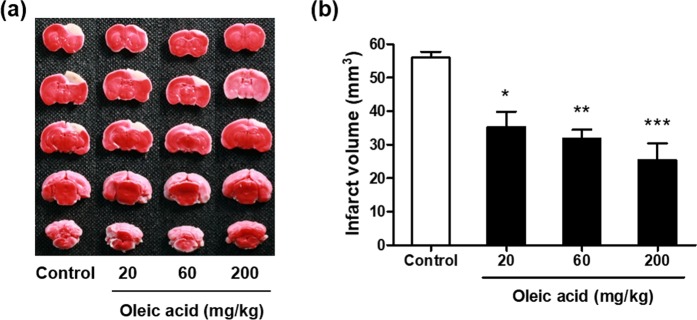
Effects of oleic acid on infarct volume after photothrombotic focal ischaemia in C57BL/6 mice. (**a**) Representative 2,3,5-triphenyl tetrazolium chloride-stained coronal sections of mouse brain. The viable tissue is stained deep red, whereas the infarcted tissue is unstained. (**b**) Quantification of the infarct volumes of each group (n = 9–11 per group). Values are the mean ± the standard error of the mean. *p < 0.05, **p < 0.01, and ***p < 0.001 vs. the vehicle-treated control group.

A focal thrombotic lesion was produced in the sensorimotor cortex of C57BL/6 mice 24 h after irradiation (Fig. 2a). Compared to the control group, which had an infarct volume of 56.1 ± 1.7 mm^3^, the intraperitoneal administration of oleic acid at doses of 20, 60, and 200 mg/kg significantly reduced the infarct volumes to 35.3 ± 4.6 mm^3^ (*p* < 0.05), 31.8 ± 2.7 mm^3^ (*p* < 0.01), and 25.4 ± 5.0 mm^3^ (*p* < 0.001), respectively (Fig. 2b).

### Effects of oleic acid on CA1 cell density after 4-VO

**Figure 3:**
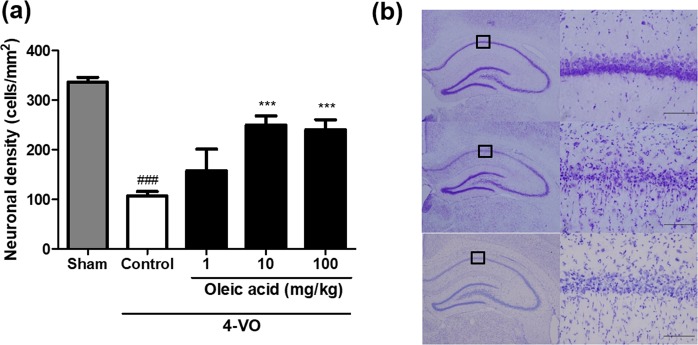
Effects of oleic acid on hippocampal neuronal damage after four-vessel occlusion (4-VO) in Wistar rats. (**a**) Quantification of neuronal densities in the hippocampal CA1 region of each group. (**b**) Representative photomicrographs of cresyl violet-stained hippocampal sections of the sham-operated group (top row), vehicle-treated control group (middle row), and oleic acid (10 mg/kg)-treated group (bottom row). Magnified images of the CA1 region in the black box in the first column are shown in the second column (×400). Scale bar = 100 μm. Values are the mean ± the standard error of the mean (n = 4–5 per group). ^###^*p* < 0.0001 vs. the sham-operated group; ***p < 0.0001 vs. the vehicle-treated control group.

Ten minutes of 4-VO resulted in massive CA1 pyramidal cell degeneration in the hippocampus of Wistar rats after 7 days, whereas oleic acid prevented this death (Fig. 3a,b). The CA1 neuronal density of the control group was significantly reduced to 106.4 ± 9.4 cells/mm^2^ compared to 335.8 ± 10.0 cells/mm^2^ in the sham-operated group (*p* < 0.001, Fig. 3a). In contrast, the intraperitoneal administration of oleic acid at doses of 10 and 100 mg/kg significantly elevated the neuronal density to 249.9 ± 18.3 cells/mm^2^ and 240.0 ± 20.5 cells/mm^2^, respectively, compared to that of the control group (both *p* < 0.001).

### Therapeutic time window of oleic acid after MCAO

**Figure 4:**
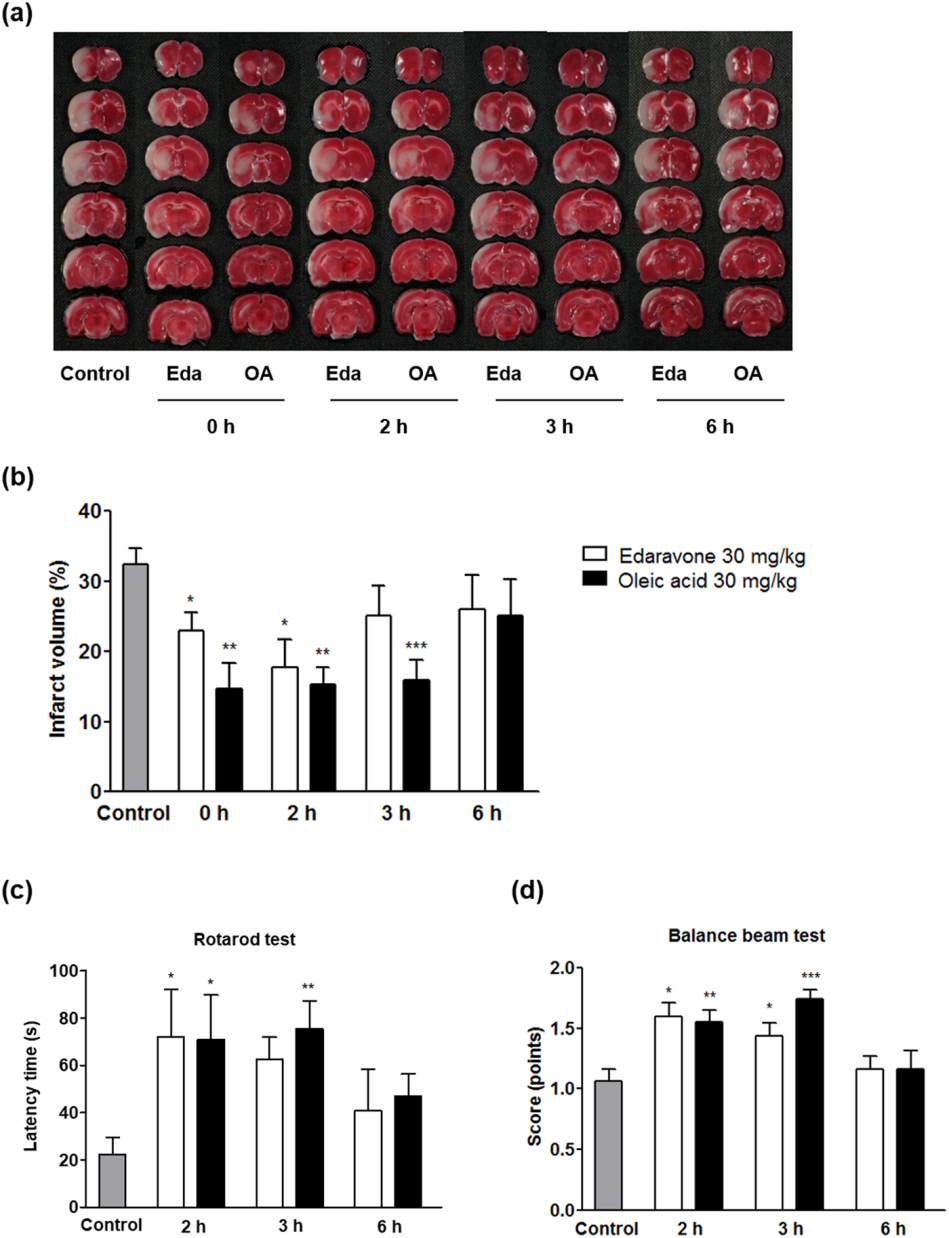
Therapeutic window of oleic acid (OA) in a Sprague-Dawley rat model of middle cerebral artery occlusion (MCAO). A single dose of OA (30 mg/kg) or edavarone (Eda; 30 mg/kg) was given intraperitoneally at different times (0, 2, 3, or 6 h) after MCAO. (**a**) Representative 2,3,5-triphenyl tetrazolium chloride-stained coronal sections of rat brain. (**b**) Quantification of infarct volumes. (**c**) Latencies to fall off the rotarod. (**d**) Balance beam scores. Values are the mean ± the standard error of the mean (n = 6–9 per group). *p < 0.05, **p < 0.01, and ***p < 0.001 vs. the control group.

The administration of oleic acid (30 mg/kg, intraperitoneally) at 0, 2, and 3 h after MCAO significantly reduced the infarct volume to 14.7 ± 3.7% (*p* < 0.01), 15.3 ± 2.5% (*p* < 0.01), and 15.9 ± 2.8% (*p* < 0.001), respectively, compared to 32.4 ± 2.2% in the control group (Fig. 4a,b). Concomitant with the reduction in infarct volume, significant improvement in the rats’ functional outcomes was observed when oleic acid was administered up to 3 h after MCAO. When administered at 2 and 3 h after MCAO, oleic acid significantly extended the latency time on the rotarod to 71.0 ± 18.8 s (*p* < 0.05) and 75.6 ± 11.6 s (*p* < 0.01), respectively, compared to 22.5 ± 7.0 s in the control group (Fig. 4c); furthermore, oleic acid significantly elevated the balance beam scores to 1.6 ± 0.1 points (*p* < 0.01) and 1.7 ± 0.1 points (*p* < 0.001), respectively, compared to 1.1 ± 0.1 points in the control group (Fig. 4d). Administering edaravone (30 mg/kg, intraperitoneally) up to 2 h after MCAO had significant beneficial effects on the infarct volume and related functional deficits.

### Effects of pre-treatment with GW9662 on oleic acid-induced neuroprotection

**Figure 5:**
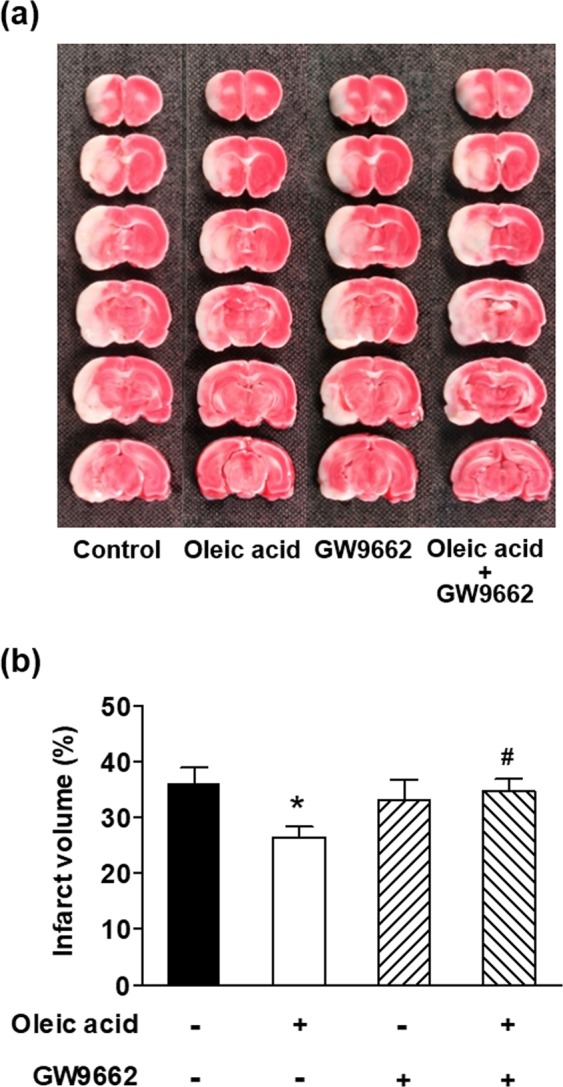
Effects of oleic acid on infarct volume is peroxisome proliferator-activated receptor gamma (PPAR-γ)-dependent in a Sprague-Dawley rat model of middle cerebral artery occlusion. The PPAR-γ antagonist GW9662 (4 mg/kg, intraperitoneally) was administered 1 h before oleic acid treatment (30 mg/kg, intraperitoneally). (**a**) Representative 2,3,5-triphenyl tetrazolium chloride-stained coronal sections of rat brain. The viable tissue is stained deep red, whereas the infarcted tissue is unstained. (**b**) Quantification of the infarct volumes of each group (n = 7–11 per group). *p < 0.05 vs. the vehicle-treated control group (first column); ^#^*p* < 0.05 vs. the oleic acid-treated group (second column).

Treatment with oleic acid (30 mg/kg, intraperitoneally) significantly reduced the infarct volume to 26.5 ± 1.9% compared to 36.0 ± 2.9% in the control group after MCAO (*p* < 0.05, Fig. 5a,b). This reduction was significantly blocked by pre-treatment with the PPAR-γ antagonist GW9662 (4 mg/kg, intraperitoneally). Treatment with GW9662 alone did not significantly affect the infarct volume.

### Effects of pre-treatment with GW9662 on oleic acid-induced inhibition of cyclooxygenase-2, inducible nitric oxide synthase, and tumour necrosis factor-alpha expression

**Figure 6:**
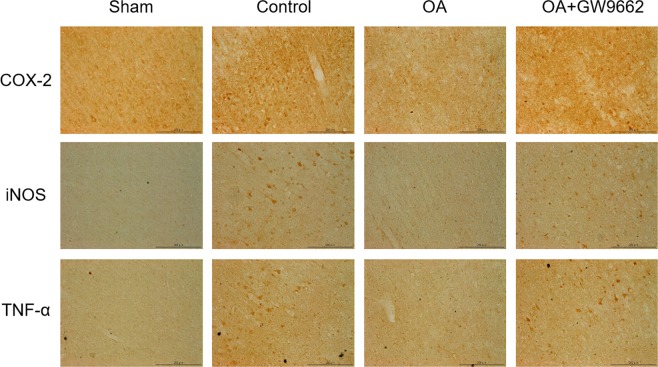
Oleic acid (OA) reduces the expression of cyclooxygenase-2 (COX-2), inducible nitric oxide synthase (iNOS), and tumour necrosis factor-alpha (TNF-α) in the peri-infarct cortex after middle cerebral artery occlusion in Sprague-Dawley rats via a peroxisome proliferator-activated receptor gamma (PPAR-γ)-dependent pathway. The PPAR-γ antagonist GW9662 (4 mg/kg, intraperitoneally) was administered 1 h before OA treatment (30 mg/kg, intraperitoneally). Representative immunostaining of coronal brain sections for COX-2, iNOS, and TNF-α (top, middle, and bottom row, respectively) from the sham-operated, vehicle-treated control, OA, and OA plus GW9662 groups (first, second, third, and fourth column, respectively). Scale bar = 200 μm.

We additionally examined whether oleic acid reduces the up-regulation of cyclooxygenase-2 (COX-2), inducible nitric oxide synthase (iNOS), and tumour necrosis factor-alpha (TNF-α) expression via a PPAR-γ-dependent mechanism. We found that the immunoreactivity of COX-2, iNOS, and TNF-α was elevated in the peri-infarct cortex of MCAO rats compared to sham-operated rats (Fig. 6). Treatment with oleic acid (30 mg/kg, intraperitoneally) markedly attenuated the immunoreactivity of COX-2, iNOS, and TNF-α compared to the vehicle-treated control group. Pre-treatment with the PPAR-γ antagonist GW9662 (4 mg/kg, intraperitoneally) reversed these inhibitory effects afforded by oleic acid.

## Discussion

The present study, to our best knowledge, is the first to show that systemic treatment with oleic acid exerted protective effects against ischaemic brain injury in both transient and permanent models of focal cerebral ischaemia, as well as in a model of transient global ischaemia. In the time window study, oleic acid was highly efficacious when administered as late as 3 h after transient focal cerebral ischaemia. Importantly, pre-treatment with GW9662, the PPAR-γ antagonist, reversed the beneficial effects of oleic acid, indicating a PPAR-γ-dependent mechanism.

All three models used in this study are well-established animal models of cerebral ischaemia and are widely used to study pharmacological neuroprotection^29^. Focal cerebral ischaemia is a condition similar to ischaemic stroke in humans. The MCAO model is the most commonly used because the majority (~70%) of human infarcts are caused by blockage of the MCA and its branches^30^. Since the presence or absence of reperfusion after an ischaemic event is one of the major variables affecting stroke outcome^31^, we adopted the MCAO model with reperfusion and additionally utilised a photothrombosis model that lacks reperfusion. The advantage of the photothrombosis model is that the vessel is occluded by platelet aggregation and local thrombi formation, which is structurally similar to the artery occlusion observed in human stroke^32^. In contrast to focal ischaemia, the 4-VO model of global ischaemia resembles the brain injury that occurs after cardiac arrest in humans^33^. The ability of oleic acid to significantly reduce neuronal damage in all three of these models indicates that oleic acid confers significant protection against ischaemic brain injury and may have robust clinical utility.

Here, oleic acid significantly reduced the MCAO-induced infarct volume, with the infarct area mainly restricted to the lateral caudoputamen and frontoparietal somatosensory cortex, known as the ischaemic core of this model^34^. After the onset of focal cerebral ischaemia, the ischaemic core rapidly proceeds to an unsalvageable infarct within minutes because of the severe decrease in cerebral blood flow followed by energy depletion, glutamate excitotoxicity, calcium overload, and consequent cell death. The tissue surrounding the ischaemic core, where the levels of cerebral blood flow are transiently above the threshold for maintaining morphological integrity (called the ischaemic penumbra), can potentially recover, and therefore is the target for neuroprotective therapy^35^. Our results indicate that oleic acid prevented ischaemic cell death in the penumbral region. Furthermore, the reduction in infarct size was accompanied by prolonged rotarod latencies and elevated balance beam scores. These two behavioural tests are both commonly used to assess motor coordination and balance alterations following MCAO in rats^36,37^. Our results suggest that oleic acid confers neuroprotection and improves sensorimotor function after transient focal ischaemia.

The photoactivation of rose bengal produces highly reactive oxygen radicals that mediate endothelial damage, followed by platelet aggregation, local clot formation, and finally occlusion of the microvasculature in a targeted region of the cortex^32^. This model induces an acute cortical infarct, which extends through the underlying cortex and subcortical white matter^38^. In the present study, the progression of neuronal injury from the infarct core to the adjacent tissue was prevented by oleic acid treatment, suggesting that oleic acid has neuroprotective effects after permanent focal cerebral ischaemia.

The present study revealed that oleic acid also significantly prevented CA1 neuronal death at 7 days after 4-VO. Transient global brain ischaemia selectively affects neurons in specific vulnerable regions of the brain, typically the hippocampal CA1 area^39^. After 5–10 min of global ischaemia, the degeneration of CA1 neurons starts on day 2–3 and spreads throughout the CA1 region by day 7^40^. The proposed mechanisms for this selective and delayed neuronal death include excitotoxicity, suppression of protein synthesis, endoplasmic reticulum stress, oxidative stress, and apoptosis^39,41^. Our findings indicate that oleic acid has protective effects against delayed neuronal death after global cerebral ischaemia.

Determination of the therapeutic time window for a given therapy is essential for its successful translation to clinical stroke. In our time window study, treatment with oleic acid at 3 h after MCAO, but not at 6 h, resulted in significant decrease of infarct volume and improved functional outcome. Considering that treatment with oleic acid at 0 and 3 h afforded similar neuroprotective effects (54.6% and 50.9%, respectively), the therapeutic window for oleic acid is estimated to be greater than 3 h and less than 6 h in our model of cerebral ischaemia. It is noteworthy that oleic acid had a wider therapeutic time window than edaravone, a neuroprotective drug for ischaemic stroke approved for use within 24 h of onset^42^. These findings indicate that oleic acid might provide a clinically feasible therapeutic window of opportunity for stroke intervention.

Several neuroprotective drugs are currently marketed for the treatment of ischaemic stroke^43^. Among these drugs, edaravone is widely used and its efficacy has been well-documented in humans and animals^44,45^, and as such, we utilised edaravone as the positive control in this study. Herein, oleic acid was found to be effective in all three models of cerebral ischaemia. In contrast, although the neuroprotection of edaravone has been confirmed in animal models of transient global and focal ischaemia, its effects on permanent focal cerebral ischaemia are controversial^46^. This is probably because edaravone is a free radical scavenger, and reperfusion causes a massive production of reactive oxygen species. Furthermore, as mentioned above, oleic acid had a broader therapeutic time window than edaravone. It is important for acute stroke therapies to have a wide therapeutic time window so that more patients can benefit from treatment. Our results suggest that oleic acid may provide more opportunities for stroke intervention.

Oleic acid is a natural ligand and activator for PPAR-γ. As an endogenous protective factor against ischaemic brain injury, PPAR-γ has been suggested as a pharmacological target for neuroprotection in cerebral ischaemia^24^. Research has shown that PPAR-γ expression rapidly increases in neurons and microglia during the first 24 h after cerebral ischaemia in rats, especially in the peri-infarct areas^47,48^, while the DNA binding of PPAR-γ and consequent target gene transcription decreases^48^. Importantly, the administration of PPAR-γ agonists has been shown to restore PPAR-γ-DNA binding activity and reduce infarct volume after cerebral ischaemia^25–28,48,49^. Various fatty acids, including oleic acid, are well-known natural ligands for PPAR-γ. PPAR-γ shows a more restricted binding profile to mono- and polyunsaturated fatty acids than saturated fatty acids^23^. Oleic acid, a monounsaturated fatty acid, has a higher PPAR-γ binding affinity than other monosaturated fatty acids, such as palmitoleic acid (C16:1) and erucic acid (C22:1), and exhibits a binding potency to PPAR-γ that is similar to that of PUFAs, which are known as strong PPAR-γ activators, along with synthetic ligands^23^. In the present study, oleic acid-induced reduction in infarct volume after MCAO was significantly abolished by the irreversible PPAR-γ antagonist GW9662, suggesting that oleic acid exerts neuroprotection against focal cerebral ischaemia through PPAR-γ activation.

Oleic acid is found in high concentration in brain membrane phospholipids, esterified at the sn-1 and -2 positions of glycerol backbone. Cerebral ischaemia induces the release of oleic acid mainly by phospholipase A_2_, leading to significant increases in free oleic acid levels in ischaemic brain regions^19,50,51^. Thus, this endogenous oleic acid might activate intracerebral PPAR-γ after cerebral ischaemia. In addition, other endogenous fatty acids released from brain membrane phospholipids after ischaemia, such as linoleic and docosahexaenoic acid, may also be agonists for PPAR-γ^19,23^. However, the infarct volume was not altered by GW9662 treatment alone in this study. GW9662 blocks ligand binding to PPAR-γ via cysteine modification in the ligand binding region of the PPAR-γ molecule but not interferes with either DNA binding or interactions with other transcription factors^52^. Hence, it does not inhibit basal PPAR-γ activity, only the ligand-mediated activation of PPAR-γ. Although one recent report demonstrated that post-ischaemic treatment with GW9662 reduced brain damage in ischaemic rats^53^, most evidence supports that pre-treatment with GW9662 alone has no significant effects on the infarct volumes or neurologic deficits in rat MCAO models^28,54^, which is consistent with the present results. Our findings suggest that endogenous PPAR-γ activity is insufficient to provide significant neuroprotection without increase by exogenous treatment.

Herein, oleic acid reduced the up-regulation of COX-2, iNOS, and TNF-α protein expression in the ischaemic brain at 24 h after MCAO. Numerous studies have confirmed the detrimental role of inflammation in neuronal damage after cerebral ischaemia^55^. Cerebral ischaemia induces the up-regulation of mRNA and protein expression of COX-2, iNOS, and TNF-α after 12–24 h in rats^56–59^. Prior research has shown that COX-2 contributes to brain damage by producing toxic prostanoids and reactive oxygen species, while iNOS generates nitric oxide in large amounts resulting in DNA damage and oxidative stress^60^. Further, TNF-α disrupts the blood-brain barrier (BBB) and consequently facilitates the influx of peripheral inflammatory cells into the injured brain^61^. These reactions exacerbate the local inflammatory responses following cerebral ischaemia. PPAR-γ agonists are known to down-regulate the expression of a variety of inflammatory genes^62^ and inhibit the post-ischaemic expression of key inflammatory genes, including COX-2, iNOS, and TNF-α, in the brain^25,47^. This is attributed, at least in part, to the ability of PPAR-γ to antagonise the activities of nuclear factor-κB (NF-κB), activator protein-1 (AP-1), and signal transducer and activator of transcription-1 (STAT1), transcription factors which play major roles in modulating the expression of inflammatory genes after cerebral ischaemia^62,63^. PPAR-γ can directly bind transcription factors, preventing them from DNA binding, or PPAR-γ can transrepress the activity of other transcription factors by binding coactivators^62^. In addition, PPAR-γ agonists have been reported to inhibit inflammatory gene expression via PPAR-γ-independent mechanisms^64,65^. In this study, the attenuation of COX-2, iNOS, and TNF-α expressions and the reduction of the infarct volume by oleic acid were abolished by the PPAR-γ antagonist GW9662, clearly indicating a PPAR-γ-dependent mechanism. In view of these findings, our results suggest that the neuroprotective effects of oleic acid might be attributable to interruption of the inflammatory response owing to the inhibition of COX-2, iNOS, and TNF-α expressions, as mediated by PPAR-γ activation.

Oleic acid is postulated to cross the BBB in its free form either by passive flip-flop diffusion or by protein-mediated facilitated uptake^66^. Transport proteins previously reported to be involved in the transport of oleic acid across the BBB include fatty acid transport protein-1 (FATP1), fatty acid translocase (FAT)/CD36, and fatty acid binding protein 5 (FABP5)^67,68^. In addition, since the BBB is disrupted in acute ischaemic stroke^69^, it might not be a significant obstacle to the entrance of oleic acid into the brain. Once it crosses the plasma membrane of neural and glial cells, free oleic acid might activate PPAR-γ and reduce the expression of post-ischaemic inflammatory mediators including COX-2, iNOS, and TNF-α in the brain.

Oleic acid is known to improve endothelial dysfunction via various pathways including the activation of PPARs^70^. It has also been reported that oleic acid inhibits endothelial activation by reducing oxidative stress, adhesion molecule expression, and monocyte adhesion in endothelial cells^71,72^, all of which could be beneficial for neuroprotection after cerebral ischaemia^73^. Based on these previous findings, it is presumed that the neuroprotective effects of oleic acid against cerebral ischaemia might be indirectly attributable to its beneficial effects on endothelial dysfunction.

The PPAR-γ agonists that have been or are currently being investigated in stroke clinical trials include antidiabetic thiazolidinediones, such as pioglitazone and rosiglitazone. In terms of safety, oleic acid is a better candidate than these drugs. Large population-based studies show that thiazolidinediones is associated with an increased risk of stroke, in addition to well-known side effects including oedema, congestive heart failure, myocardial infarction, bladder cancer, and fractures^74,75^. In contrast, oleic acid has been widely consumed, as a major component of vegetable oils and animal fats, on a daily basis and has not been reported to cause significant adverse effects. Furthermore, a previous cohort study demonstrated that a higher plasma level of oleic acid was associated with a lower stroke incidence^76^.

Oleic acid is a large constituent (up to 80%) of olive oil^77^. Olive oil is known to provide neuroprotection against various neurological disorders^78,79^. In an experimental model of stroke, olive oil reduced the infarct volume and neurological deficits when administered after ischaemia^80^. The major contributors to this neuroprotection have generally been considered to be olive oil phenols including oleuropein, hydroxytyrosol, and tyrosol^78,79^. Our findings provide new evidence that oleic acid may also contribute to the olive oil-mediated neuroprotection, along with the phenolic components.

One of the limitations of this study is that the origin of brain oleic acid after ischaemia is unclear. As mentioned earlier, oleic acid is released from brain membrane phospholipids primarily by phospholipase A_2_ during ischaemia^19,50,51^. It can also be endogenously produced from triglycerides by lipolysis, released into the circulatory system, and delivered to the brain bound to albumin^81^. Another limitation is that oleic acid level in the plasma or brain were not measured before and after the treatment, and thus the level of exogenously administered free oleic acid does not clearly indicate the levels of total oleic acid. In addition, we did not trace the incorporation of administered oleic acid in brain phospho-/neutral lipids. It has been reported that phospholipids also serve as PPAR-γ ligands, although their binding affinity is relatively low^82^. Thus, it is not clear whether oleic acid either as a free fatty acid or as a constituent of lipids (or as both) interacts with PPAR-γ. Further studies such as lipid tracer studies are needed to reveal the origin and fate of oleic acid in the ischaemic brain.

In summary, our findings support that oleic acid exerts neuroprotective effects in transient and permanent focal cerebral ischaemia, as well as protective effects against global cerebral ischaemia-induced delayed neuronal death. In addition, oleic acid improves sensorimotor function with a clinically feasible therapeutic window after transient focal cerebral ischaemia. As such, it may be of therapeutic value for the treatment of ischaemic brain injuries, such as ischaemic stroke. The oleic acid-mediated neuroprotection we observed might be attributable to its anti-inflammatory actions, as regulated by PPAR-γ activation.

## Materials and Methods

### Animals

Male SD rats (300 ± 10 g), male C57BL/6 mice (25 ± 1 g), and male Wistar rats (180 ± 10 g) were purchased from Samtako Inc. (Osan, Korea). Animals were housed in a facility with constant humidity (60 ± 10%), temperature (23 ± 1 °C), and a 12-h light/dark cycle (lights on at 7 am). Food and water were available *ad libitum* throughout the study. Animals were acclimatised to laboratory conditions for at least 1 week before undergoing surgery. Analgesics, such as opioids and nonsteroidal anti-inflammatory drugs, were not administered during the postoperative phase because they may interfere with the assessment of the neuroprotective therapies^83^. All experimental procedures were performed according to the Principles of Laboratory Animal Care (National Institutes of Health publication, #85–23, revised in 1985). The experimental protocols were approved the Institutional Animal Care and Use Committee of Korea Institute of Science and Technology for Eastern Medicine (approval no. KISTEM-IACUC-2017-004).

### MCAO model

Transient focal cerebral ischaemia was induced in male SD rats by MCAO using the method of Zea-Longa *et al*.^84^. We employed SD rats because they are the most widely used strain in transient MCAO experiments^85^. Briefly, rats were anesthetised with isoflurane (induction with 5% isoflurane and maintenance with 2% isoflurane) in N_2_O/O_2_ (7:3). The bifurcation of the left common carotid artery was exposed through a midline incision in the neck. A silicon rubber-coated monofilament (360 ± 5 μm in diameter) was inserted into the external carotid artery and advanced to18–20 mm from the carotid bifurcation to occlude the MCA origin. After 90 min, the monofilament was withdrawn to allow reperfusion. Rectal temperature was maintained at 37 ± 0.5 °C using a heating lamp and a homoeothermic blanket system (Harvard Apparatus, USA) throughout the surgery. Occlusion of the MCA was confirmed by the presence of characteristic behavioural deficits, such as paralysed forelimb flexion, torso twist, and spontaneous circling after reperfusion^86,87^. Rats that did not meet these criteria were excluded from the experiment.

### Photothrombosis model

Permanent focal cerebral ischaemia was induced in male C57BL/6 mice by photothrombosis of the cortical microvessels as described previously^88^ with slight modifications. C57BL/6 mice were selected because this strain is widely used in rose bengal-induced cold-light photothrombotic occlusion experiments^88,89^. Briefly, mice were anesthetised by injecting chloral hydrate (450 mg/kg). Rose bengal solution (100 μL, 10 mg/mL in sterile saline; Sigma, USA) was administered intraperitoneally 5 min before illumination. Mice were positioned in a stereotaxic frame and the skull was exposed via a midline scalp incision. Following this, a fibre-optic bundle of a cold white light source with a 4-mm aperture (CL 6000 LED; Carl Zeiss, Germany) was placed 2 mm lateral from bregma. The brain was then illuminated for 15 min through the intact exposed skull. Thereafter, the skin was sutured, and mice were allowed to recover. Rectal temperature was kept at 37 ± 0.5 °C using a heating pad during the surgery.

### 4-VO model

Transient global cerebral ischaemia was induced in male Wistar rats using the 4-VO model described by Pulsinelli and Brierly^90^. Wistar rats were chosen, as this strain is known to have a higher rate of successful global ischaemia than the other strains^33^. Briefly, rats were anesthetised with isoflurane as described above, and the vertebral arteries were electrocauterised. The left and right common carotid arteries were carefully isolated using loops of thread. On the following day, both common carotid arteries were occluded with aneurysm clips for 10 min to induce global cerebral ischaemia and then the aneurysm clips were removed for reperfusion. Criteria for successful 4-VO included the loss of the righting reflex, bilateral pupil dilation, and unresponsiveness to tail pinch throughout the ischaemic period. To minimise variability, rats with 20 ± 5 min of post-ischaemic coma were included in the experiment^91^. Rats that developed seizure activity during or after ischaemia were excluded from the experiment. Rectal temperature was controlled at 37 ± 0.5 °C until 6 h after ischaemia.

### Experimental design and sample treatment

In all experiments, oleic acid (Sigma, USA) and edaravone (Mitsubishi Pharma Co., Japan) were dissolved in 5% Tween^®^ 20 (Sigma, USA) in sterile saline and administered intraperitoneally. First, we examined the efficacy of oleic acid administration in each model of ischaemic stroke. In the MCAO model, rats were treated with vehicle (control), 10 mg/kg of oleic acid, 30 mg/kg of oleic acid, or 30 mg/kg of edaravone at 0 and 90 min after MCAO. In the preliminary experiments, we also tested a dose of 100 mg/kg, and the neuroprotective effects at that dose were similar to those observed with the 30 mg/kg dosing protocol (see Fig. S1). Thus, the doses in the following experiments were set at 10 and 30 mg/kg. Rotarod and balance beam tests were conducted at 20 and 22 h after ischaemia, respectively, and then infarct volume was measured by 2,3,5-triphenyltetrazolium chloride (TTC; Sigma, USA) staining after 24 h of ischaemia. In the photothrombosis model, mice were administered twice the dose used in the MCAO rat model, i.e. 20, 60, and 200 mg/kg. This is because, when the surface area to weight ratio of the two species is considered, the mouse dose is equivalent to about twice the rat dose^92^. Oleic acid was administered in a single intraperitoneal dose following illumination, and infarct volume was measured by TTC staining at 24 h after ischaemia. In the 4-VO model, doses of 1, 10, and 100 mg/kg were selected to evaluate dose-response effects at lower doses than those used in the MCAO model. Through years of experiments, our group has found that, if a substance exerts neuroprotective effects in both the 4-VO and MCAO models, its effective doses are usually lower in the 4-VO model than in the MCAO model. For instance, in the case of *Scutellaria baicalensis* extract, the effective dose in the MCAO model was 100 mg/kg, whereas it showed neuroprotective effects at a dose as low as 0.1 mg/kg in the 4-VO model^93,94^. Oleic acid was administered immediately after reperfusion and sham-operated rats were administered vehicle with the same regimen. The neuronal density in the CA1 region of hippocampus was measured using cresyl violet staining at 7 days after ischaemia.

To determine the therapeutic time window, oleic acid or edaravone was administered in a single dose of 30 mg/kg at 0, 2, 3, or 6 h after MCAO. Rotarod and balance beam tests were conducted at 20 and 22 h after ischaemia, respectively, and then infarct volume was measured by TTC staining at 24 h after ischaemia.

We next investigated the relevance of PPAR-γ activation to the neuroprotective effects of oleic acid in the MCAO model. Rats were randomly assigned to the following four groups: control, oleic acid, GW9661, and oleic acid +GW9661. Oleic acid (30 mg/kg) was dissolved in 5% Tween^®^ 20 in sterile saline and administered intraperitoneally at 0 and 90 min after MCAO. GW9662 (4 mg/kg) was dissolved in 5% dimethylsulfoxide in sterile saline and administered intraperitoneally at 1 h before MCAO. The control group received vehicle in place of both oleic acid and GW9662, the oleic acid group received oleic acid (30 mg/kg) and vehicle instead of GW9662, the GW9661 group received vehicle rather than oleic acid and GW9662 (4 mg/kg), and the oleic acid +GW9661 group received oleic acid (30 mg/kg) and GW9662 (4 mg/kg). Infarct volume was measured at 24 h after ischaemia.

We additionally examined whether oleic acid attenuates COX-2, iNOS, and TNF-α expression through PPAR-γ activation in the MCAO model using immunohistochemistry. Rats were randomly assigned to the following four groups: sham, vehicle control, oleic acid, and oleic acid +GW9661. Oleic acid and GW9662 were administered as described above. Rats were sacrificed at 24 h after ischaemia, and brains were collected for immunohistochemical analyses.

### Measurement of infarct volume

Brains were rapidly removed, frozen, and cut into 2-mm coronal sections. Slices were then immersed in a saline solution containing 2% TTC (Sigma, USA) at 37 °C for 30 min. Slices were photographed using a digital camera and quantified using Image Pro Plus 5.1 software (Media Cybernetics, USA). The infarct volume is calculated by multiplying the lesion area by the section thickness. In the MCAO model, the infarct volume is expressed as a percent of the contralesional hemisphere volume to correct for brain oedema.

### Cresyl violet staining and measurement of neuronal density

Rats were anesthetised and perfused transcardially with heparinised 0.5% sodium nitrite saline followed by 4% paraformaldehyde. Brains were removed, fixed, and cut into 30-μm coronal sections on a sliding microtome (Microm HM 440E; Microm International, Germany). Sections were stained with cresyl violet and neuronal density of the hippocampal CA1 region was measured as per the method described in our previous study^91^. Briefly, viable cells in 6 frames (1.0 mm × 1.0 mm) of the left and right CA1 areas of 3 sections, which were located approximately 3.3, 3.5, and 3.7 mm caudal to bregma, were measured at a magnification of ×400 for each rat. The neuronal densities are expressed as the mean number of viable neurons per frame.

### Rotarod test

Rats were placed onto an accelerating rotarod (from 0 to 25 rpm; Ugo Basile, Milan, Italy) and tested for 2 min. For each rat, latency times were recorded in 5 separate trials. The highest and lowest values were excluded and the mean of the remaining 3 trial results was used for the analysis.

### Balance beam test

Balance beam testing was conducted as per a previous method with slight modifications37. Briefly, rats were positioned on the centre of a square beam (2.5 cm × 122 cm × 42 cm). Their performance on the beam was rated on a scale from 0 to 6 points, as follows: 0 = rats were not able to stay; 1 = rats were able to stay, but did not move; 2 = rats attempted to cross the beam, but fell; 3 = rats crossed the beam with greater than 50% foot slips of the injured hindlimb; 4 = rats crossed the beam with greater than one foot slip, but less than 50%; 5 = rats crossed the beam with only one foot slip; and 6 = rats crossed the beam without any foot slips.

### Immunohistochemistry

Rats were anesthetised and perfused transcardially with heparinised 0.5% sodium nitrite saline followed by 4% paraformaldehyde. Brains were quickly removed, fixed, and cut into 40-μm coronal sections using a cryostat (Leica, Germany). Free-floating sections were reacted with rabbit polyclonal antibodies against COX-2 (1:100; Abcam, UK), iNOS (1:100; Abcam), or TNF-α (1:100; Abcam) overnight at room temperature. Subsequently, the sections were reacted with biotinylated rabbit antibody (1:200; Sigma, USA), and incubated with avidin-biotin complex reagent (Vector Laboratories, USA) for 1 h. The sections were visualised with 0.05% 3,3-diaminobenzidine solution (Sigma, USA) containing hydrogen peroxide.

### Statistical analysis

All data are presented as the mean ± the standard error of the mean. Differences among the groups were evaluated by one-way analyses of variance followed by Dunnett’s tests using GraphPad Prism 5 (GraphPad Software Inc., USA). Statistical significance was set at *p* < 0.05.

## Supplementary information

Figure S1

## Data Availability

The data that support the findings of this study are available from the corresponding author on reasonable request.

## References

[1] McNamara RK, Carlson SE (2006). Role of omega-3 fatty acids in brain development and function: potential implications for the pathogenesis and prevention of psychopathology. Prostaglandins Leukot. Essent. Fatty Acids.

[2] Rioux FM, Innis SM (1992). Oleic acid (18:1) in plasma, liver and brain myelin lipid of piglets fed from birth with formulas differing in 18:1 content. J. Nutr..

[3] Martínez M, Mougan I (1998). Fatty acid composition of human brain phospholipids during normal development. J. Neurochem..

[4] Velasco A, Tabernero A, Medina JM (2003). Role of oleic acid as a neurotrophic factor is supported *in vivo* by the expression of GAP-43 subsequent to the activation of SREBP-1 and the up-regulation of stearoyl-CoA desaturase during postnatal development of the brain. Brain Res..

[5] Rodríguez-Rodríguez RA, Tabernero A, Velasco A, Lavado EM, Medina JM (2004). The neurotrophic effect of oleic acid includes dendritic differentiation and the expression of the neuronal basic helix-loop-helix transcription factor NeuroD2. J. Neurochem..

[6] Polo-Hernández E, De Castro F, García-García AG, Tabernero A, Medina JM (2010). Oleic acid synthesized in the periventricular zone promotes axonogenesis in the striatum during brain development. J. Neurochem..

[7] Polo-Hernández E (2014). Oleic acid synthesized by stearoyl-CoA desaturase (SCD-1) in the lateral periventricular zone of the developing rat brain mediates neuronal growth, migration and the arrangement of prospective synapses. Brain Res..

[8] Martín V (2010). Lipid alterations in lipid rafts from Alzheimer’s disease human brain cortex. J. Alzheimers Dis..

[9] Hamazaki K, Hamazaki T, Inadera H (2012). Fatty acid composition in the postmortem amygdala of patients with schizophrenia, bipolar disorder, and major depressive disorder. J. Psychiatr. Res..

[10] Amtul Z, Westaway D, Cechetto DF, Rozmahel RF (2011). Oleic acid ameliorates amyloidosis in cellular and mouse models of Alzheimer’s disease. Brain Pathol..

[11] Debbabi M (2016). Protective Effects of α-tocopherol, γ-tocopherol and oleic acid, three compounds of olive oils, and no effect of trolox, on 7-ketocholesterol-induced mitochondrial and peroxisomal dysfunction in microglial BV-2 cells. Int. J. Mol. Sci..

[12] Debbabi M (2017). Comparison of the effects of major fatty acids present in the Mediterranean diet (oleic acid, docosahexaenoic acid) and in hydrogenated oils (elaidic acid) on 7-ketocholesterol-induced oxiapoptophagy in microglial BV-2 cells. Chem. Phys. Lipids.

[13] Dhillon HS, Dose JM, Scheff SW, Prasad MR (1997). Time course of changes in lactate and free fatty acids after experimental brain injury and relationship to morphologic damage. Exp. Neurol..

[14] Lewis MD, Bailes J (2011). Neuroprotection for the warrior: dietary supplementation with omega-3 fatty acids. Mil. Med..

[15] Blondeau N (2015). Alpha-linolenic acid: an omega-3 fatty acid with neuroprotective properties-ready for use in the stroke clinic?. Biomed Res. Int..

[16] Qu Y, Zhang HL, Zhang XP, Jiang HL (2018). Arachidonic acid attenuates brain damage in a rat model of ischemia/reperfusion by inhibiting inflammatory response and oxidative stress. Hum. Exp. Toxicol..

[17] Rink C, Khanna S (2011). Significance of brain tissue oxygenation and the arachidonic acid cascade in stroke. Antioxid. Redox. Signal..

[18] Hennebelle M (2017). Linoleic acid participates in the response to ischemic brain injury through oxidized metabolites that regulate neurotransmission. Sci. Rep..

[19] Pilitsis JG (2003). Measurement of free fatty acids in cerebrospinal fluid from patients with hemorrhagic and ischemic stroke. Brain Res..

[20] Lee DH (2006). Neuroprotective effect of Buddleja officinalis extract on transient middle cerebral artery occlusion in rats. Biol. Pharm. Bull..

[21] Oh YT (2009). Oleic acid reduces lipopolysaccharide-induced expression of iNOS and COX-2 in BV2 murine microglial cells: possible involvement of reactive oxygen species, p38 MAPK, and IKK/NF-κB signaling pathways. Neurosci. Lett..

[22] Weinstein JR, Koerner IP, Möller T (2010). Microglia in ischemic brain injury. Future Neurol..

[23] Xu HE (1999). Molecular recognition of fatty acids by peroxisome proliferator-activated receptors. Mol. Cell.

[24] Culman J, Zhao Y, Gohlke P, Herdegen T (2007). PPAR-γ: therapeutic target for ischemic stroke. Trends Pharmacol. Sci..

[25] Sundararajan S (2005). Peroxisome proliferator-activated receptor-γ ligands reduce inflammation and infarction size in transient focal ischemia. Neuroscience.

[26] Pereira MP (2005). The nonthiazolidinedione PPARγ agonist L-796,449 is neuroprotective in experimental stroke. J. Neuropathol. Exp. Neurol..

[27] Lin TN (2006). 15d-prostaglandin J_2_ protects brain from ischemia-reperfusion injury. Arterioscler. Thromb. Vasc. Biol..

[28] Zhang HL (2011). Neuroprotective effects of pioglitazone in a rat model of permanent focal cerebral ischemia are associated with peroxisome proliferator-activated receptor gamma-mediated suppression of nuclear factor-κB signaling pathway. Neuroscience.

[29] Bacigaluppi M, Comi G, Hermann DM (2010). Animal models of ischemic stroke. Part two: modeling cerebral ischemia. Open Neurol. J..

[30] Bogousslavsky J, Melle GV, Regli F (1988). The Lausanne Stroke Registry: analysis of 1,000 consecutive patients with first stroke. Stroke.

[31] Warach S, Latour LL (2004). Evidence of reperfusion injury, exacerbated by thrombolytic therapy, in human focal brain ischemia using a novel imaging marker of early blood-brain barrier disruption. Stroke.

[32] Labat-gest, V. & Tomasi, S. Photothrombotic ischemia: a minimally invasive and reproducible photochemical cortical lesion model for mouse stroke studies. *J*. *Vis*. *Exp*. **76**, 10.3791/50370 (2013). 10.3791/50370 PMC3727176 23770844

[33] Deng, P. & Xu, Z. C. Four-vessel occlusion model in rats. In *Animal models of acute neurological injuries* (eds Chen, J., Xu, Z. C., Xu, X. M. & Zhang, J. H.) 65–76 (Humana Press, 2009).

[34] Belayev L, Zhao W, Busto R, Ginsberg MD (1997). Transient middle cerebral artery occlusion by intraluminal suture: I. Three-dimensional autoradiographic image-analysis of local cerebral glucose metabolism-blood flow interrelationships during ischemia and early recirculation. J. Cereb. Blood Flow Metab..

[35] Heiss WD (2000). Ischemic penumbra: evidence from functional imaging in man. J. Cereb. Blood Flow Metab..

[36] Zhang L, Chen J, Li Y, Zhang ZG, Chopp M (2000). Quantitative measurement of motor and somatosensory impairments after mild (30 min) and severe (2 h) transient middle cerebral artery occlusion in rats. J. Neurol. Sci..

[37] Puurunen K, Jolkkonen J, Sirviö J, Haapalinna A, Sivenius J (2001). An α_2_-adrenergic antagonist, atipamezole, facilitates behavioral recovery after focal cerebral ischemia in rats. Neuropharmacology.

[38] Eichenbaum JW (2002). A murine photochemical stroke model with histologic correlates of apoptotic and nonapoptotic mechanisms. J. Pharmacol. Toxicol. Methods.

[39] Schmidt-Kastner R (2015). Genomic approach to selective vulnerability of the hippocampus in brain ischemia-hypoxia. Neuroscience.

[40] Lipton P (1999). Ischemic cell death in brain neurons. Physiol. Rev..

[41] Yigitkanli K, Zheng Y, Pekcec A, Lo EH, van Leyen K (2017). Increased 12/15-lipoxygenase leads to widespread brain injury following global cerebral ischemia. Transl. Stroke Res..

[42] Edaravone Acute Infarction Study Group (2003). Effect of a novel free radical scavenger, edaravone (MCI-186), on acute brain infarction. Randomized, placebo-controlled, double-blind study at multicenters. Cerebrovasc. Dis..

[43] Karsy M (2017). Neuroprotective strategies and the underlying molecular basis of cerebrovascular stroke. Neurosurg. Focus.

[44] Yang J (2015). Edaravone for acute stroke: Meta-analyses of data from randomized controlled trials. Dev. Neurorehabil..

[45] Ren Y (2015). Edaravone’s free radical scavenging mechanisms of neuroprotection against cerebral ischemia: review of the literature. Int. J. Neurosci..

[46] Takamatsu H, Kondo K, Ikeda Y, Umemura K (1998). Neuroprotective effects depend on the model of focal ischemia following middle cerebral artery occlusion. Eur. J. Pharmacol..

[47] Zhao Y, Patzer A, Herdegen T, Gohlke P, Culman J (2006). Activation of cerebral peroxisome proliferator-activated receptors gamma promotes neuroprotection by attenuation of neuronal cyclooxygenase-2 overexpression after focal cerebral ischemia in rats. FASEB J..

[48] Victor NA (2006). Altered PPARγ expression and activation after transient focal ischemia in rats. Eur. J. Neurosci..

[49] Ou Z (2006). Neuronal expression of peroxisome proliferator-activated receptor-gamma (PPARγ) and 15d-prostaglandin J_2_—Mediated protection of brain after experimental cerebral ischemia in rat. Brain Res..

[50] Phillis JW, O’Regan MH (2004). A potentially critical role of phospholipases in central nervous system ischemic, traumatic, and neurodegenerative disorders. Brain Res. Rev..

[51] Lopez S (2014). Membrane composition and dynamics: a target of bioactive virgin olive oil constituents. Biochim. Biophys. Acta..

[52] Leesnitzer LM (2002). Functional consequences of cysteine modification in the ligand binding sites of peroxisome proliferator activated receptors by GW9662. Biochemistry.

[53] Lin Chi-Hsin, Liao Li-Ya, Yang Tsung-Ying, Chang Yi-Jyun, Tung Chia-Wen, Hsu Shih-Lan, Hsueh Chi-Mei (2019). Microglia-Derived Adiposomes are Potential Targets for the Treatment of Ischemic Stroke. Cellular and Molecular Neurobiology.

[54] Shan BS (2018). Attenuation of stroke damage by angiotensin II type 2 receptor stimulation via peroxisome proliferator-activated receptor-gamma activation. Hypertens. Res..

[55] Kawabori M, Yenari MA (2015). Inflammatory responses in brain ischemia. Curr. Med. Chem..

[56] Nogawa S, Zhang F, Ross ME, Iadecola C (1997). Cyclo-oxygenase-2 gene expression in neurons contributes to ischemic brain damage. J. Neurosci..

[57] Iadecola C, Zhang F, Xu S, Casey R, Ross ME (1995). Inducible nitric oxide synthase gene expression in brain following cerebral ischemia. J. Cereb. Blood Flow Metab..

[58] Iadecola C, Zhang F, Casey R, Clark HB, Ross ME (1996). Inducible nitric oxide synthase gene expression in vascular cells after transient focal cerebral ischemia. Stroke.

[59] Liu T (1994). Tumor necrosis factor-α expression in ischemic neurons. Stroke.

[60] Iadecola C, Alexander M (2001). Cerebral ischemia and inflammation. Curr. Opin. Neurol..

[61] Watters O, O’Connor JJ (2011). A role for tumor necrosis factor-α in ischemia and ischemic preconditioning. J. Neuroinflammation.

[62] Daynes RA, Jones DC (2002). Emerging roles of PPARs in inflammation and immunity. Nat. Rev. Immunol..

[63] Harari OA, Liao JK (2010). NF-κB and innate immunity in ischemic stroke. Ann. N. Y. Acad. Sci..

[64] Li M, Pascual G, Glass CK (2000). Peroxisome proliferator-activated receptor γ-dependent repression of the inducible nitric oxide synthase gene. Mol. Cell. Biol..

[65] Chawla A (2001). PPAR-γ dependent and independent effects on macrophage-gene expression in lipid metabolism and inflammation. Nat Med..

[66] Tracey TJ, Steyn FJ, Wolvetang EJ, Ngo ST (2018). Neuronal lipid metabolism: multiple pathways driving functional outcomes in health and disease. Front. Mol. Neurosci..

[67] Mitchell RW, Edmundson CL, Miller DW, Hatch GM (2009). On the mechanism of oleate transport across human brain microvessel endothelial cells. J. Neurochem..

[68] Mitchell RW, On NH, Del Bigio MR, Miller DW, Hatch GM (2011). Fatty acid transport protein expression in human brain and potential role in fatty acid transport across human brain microvessel endothelial cells. J. Neurochem..

[69] Ballabh P, Braun A, Nedergaard M (2004). The blood-brain barrier: an overview: structure, regulation, and clinical implications. Neurobiol. Dis..

[70] Ringseis R, Eder K (2010). Fatty acids and signalling in endothelial cells. Prostaglandins Leukot. Essent. Fatty Acids..

[71] Carluccio MA (1999). Oleic acid inhibits endothelial activation: A direct vascular antiatherogenic mechanism of a nutritional component in the mediterranean diet. Arterioscler. Thromb. Vasc. Biol..

[72] Massaro M (2002). Quenching of intracellular ROS generation as a mechanism for oleate-induced reduction of endothelial activation and early atherogenesis. Thromb. Haemost..

[73] Lakhan SE, Kirchgessner A, Hofer M (2009). Inflammatory mechanisms in ischemic stroke: therapeutic approaches. J. Transl. Med..

[74] Kung J, Henry RR (2012). Thiazolidinedione safety. Expert Opin. Drug Saf..

[75] Lu CJ (2013). Risk of stroke with thiazolidinediones: a ten-year nationwide population-based cohort study. Cerebrovasc. Dis..

[76] Samieri C (2011). Olive oil consumption, plasma oleic acid, and stroke incidence: the Three-City Study. Neurology.

[77] Beltrán G, Del Rio C, Sánchez S, Martínez L (2004). Influence of harvest date and crop yield on the fatty acid composition of virgin olive oils from cv. Picual. J. Agric. Food Chem..

[78] Khalatbary AR (2013). Olive oil phenols and neuroprotection. Nutr. Neurosci..

[79] Angeloni C, Malaguti M, Barbalace MC, Hrelia S (2017). Bioactivity of olive oil phenols in neuroprotection. Int. J. Mol. Sci..

[80] Sarshoori JR, Asadi MH, Mohammadi MT (2014). Effect of olive oil on the cerebral reperfusion following ischemia injuries in rat. J. Birjand Univ. Med. Sci..

[81] Teusink B (2003). Contribution of fatty acids released from lipolysis of plasma triglycerides to total plasma fatty acid flux and tissue-specific fatty acid uptake. Diabetes.

[82] Harmon GS, Lam MT, Glass CK (2011). PPARs and lipid ligands in inflammation and metabolism. Chem. Rev..

[83] Pétrault M (2017). Neither nefopam nor acetaminophen can be used as postoperative analgesics in a rat model of ischemic stroke. Fundam. Clin. Pharmacol..

[84] Longa EZ, Weinstein PR, Carlson S, Cummins R (1989). Reversible middle cerebral artery occlusion without craniectomy in rats. Stroke.

[85] Dittmar MS (2006). Fischer-344 rats are unsuitable for the MCAO filament model due to their cerebrovascular anatomy. J. Neurosci. Methods.

[86] Park S (2012). Forced exercise enhances functional recovery after focal cerebral ischemia in spontaneously hypertensive rats. Brain Sci..

[87] Rewell SS (2017). Evolution of ischemic damage and behavioural deficit over 6 months after MCAo in the rat: Selecting the optimal outcomes and statistical power for multi-centre preclinical trials. PLoS One.

[88] Schroeter M, Jander S, Stoll G (2002). Non-invasive induction of focal cerebral ischemia in mice by photothrombosis of cortical microvessels: characterization of inflammatory responses. J. Neurosci. Methods.

[89] Park SK (2006). Photochemically induced cerebral ischemia in a mouse model. J. Korean Neurosurg. Soc..

[90] Pulsinelli WA, Brierley JB (1979). A new model of bilateral hemispheric ischemia in the unanesthetized rat. Stroke.

[91] Lee D (2012). Neuroprotective effects of *Eleutherococcus senticosus* bark on transient global cerebral ischemia in rats. J. Ethnopharmacol..

[92] Nair AB, Jacob S (2016). A simple practice guide for dose conversion between animals and human. J. Basic Clin. Pharm..

[93] Kim YO (2001). Cytoprotective effect of *Scutellaria baicalensis* in CA1 hippocampal neurons of rats after global cerebral ischemia. J. Ethnopharmacol..

[94] Gaire BP (2012). Neuroprotective effect of *Scutellaria baicalensis* against MCAo induced focal cerebral ischeinia. JHAS.

