# Supplementary material for: Neuroprotective effects of oleic acid in rodent models of cerebral ischaemia
Source: Sci Rep. 2019 Jul 24;9:10732. doi: 10.1038/s41598-019-47057-z (PMC6656890; doi:10.1038/s41598-019-47057-z)
Supplement: Supplementary file 1 — Figure S1 [file 41598_2019_47057_MOESM1_ESM.docx]

**Supplementary information**

**Neuroprotective effects of oleic acid in rodent models of cerebral ischaemia**

**Jungbin Song^1^, Dong Hwan Lee^1^, Sung Hyun Lee^2^, Hyo Jin Park^2^, Hocheol Kim^1^, Young-Sik Kim^1,*^, Donghun Lee^3,*^**

^1^Department of Herbal Pharmacology, College of Korean Medicine, Kyung Hee University, 26 Kyungheedae-ro, Dongdaemun-gu, Seoul 02447, Republic of Korea

^2^Korea Institute of Science and Technology for Eastern Medicine (KISTEM) NeuMed Inc., 88 Imun-ro, Dongdaemun-gu, Seoul 02440, Republic of Korea

^3^Department of Herbal Pharmacology, College of Korean Medicine, Gachon University, 1342 Seongnamdae-ro, Sujeong-gu, Seongnam-si, Gyeonggi-do 13120, Republic of Korea


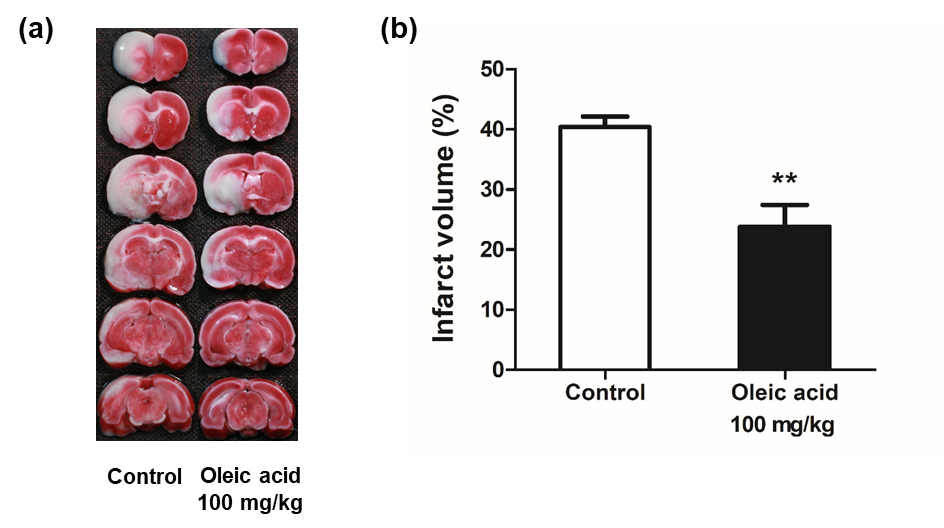


**Figure S1.** Effect of oleic acid (100 mg/kg) on infarct volume after middle cerebral artery occlusion in Sprague-Dawley rats. (**a**) Representative 2,3,5-triphenyl tetrazolium chloride-stained coronal sections of rat brain. The viable tissue is stained deep red, whereas the infarcted tissue is unstained. (**b**) Quantification of infarct volume. The administration of oleic acid (100 mg/kg) significantly reduced the infarct volume to 23.8 ± 3.6% (*p* < 0.01) compared to 40.4 ± 1.7% in the control group. ***p* < 0.01 *vs*. the vehicle-treated control group by Student’s *t*-test. Values are the mean ± the standard error of the mean (n = 7 and 5 for the control and oleic acid 100 mg/kg groups, respectively).
